# Interaction of oxalic acid with methylamine and its atmospheric implications†

**DOI:** 10.1039/c7ra13670f

**Published:** 2018-02-14

**Authors:** Yu Hong, Yi-Rong Liu, Hui Wen, Shou-Kui Miao, Teng Huang, Xiu-Qiu Peng, Shuai Jiang, Ya-Juan Feng, Wei Huang

**Affiliations:** Laboratory of Atmospheric Physico-Chemistry, Anhui Institute of Optics & Fine Mechanics, Chinese Academy of Sciences Hefei Anhui 230031 China; School of Information Science and Technology, University of Science and Technology of China Hefei Anhui 230026 China; CAS Center for Excellence in Urban Atmospheric Environment, Institute of Urban Environment, Chinese Academy of Sciences Xiamen Fujian 361021 China huangwei6@ustc.edu.cn

## Abstract

Oxalic acid, which is one of the most common dicarboxylic acids, is expected to be an important component of atmospheric aerosols. However, the contribution of oxalic acid to the generation of new particles is still poorly understood. In this study, the structural characteristics and thermodynamics of (C_2_H_2_O_4_)(CH_3_NH_2_)_*n*_ (*n* = 1–4) were investigated at the PW91PW91/6-311++G(3df,3pd) level of theory. We found that clusters formed by oxalic acid and methylamine are relatively stable, and the more the atoms participating in the formation of a ring-like structure, the more stable is the cluster. In addition, *via* the analysis of atmospheric relevance, it can be revealed that clusters of (C_2_H_2_O_4_)(CH_3_NH_2_)_*n*_ (*n* = 1–4) have a noteworthy concentration in the atmosphere, which indicates that these clusters could be participating in new particle formation. Moreover, by comparison with (H_2_C_2_O_4_)(NH_3_)_*n*_ (*n* = 1–6) species, it can be seen that oxalic acid is more readily bound to methylamine than to ammonia, which promotes nucleation or new particle formation. Finally, the Rayleigh scattering properties of clusters of (C_2_H_2_O_4_)(CH_3_NH_2_)_*n*_ (*n* = 1–4) were investigated for the first time to determine their atmospheric implications.

## Abstract 2

Oxalic acid, which is one of the most common dicarboxylic acids, is expected to be an important component of atmospheric aerosols.

## Graphical abstract

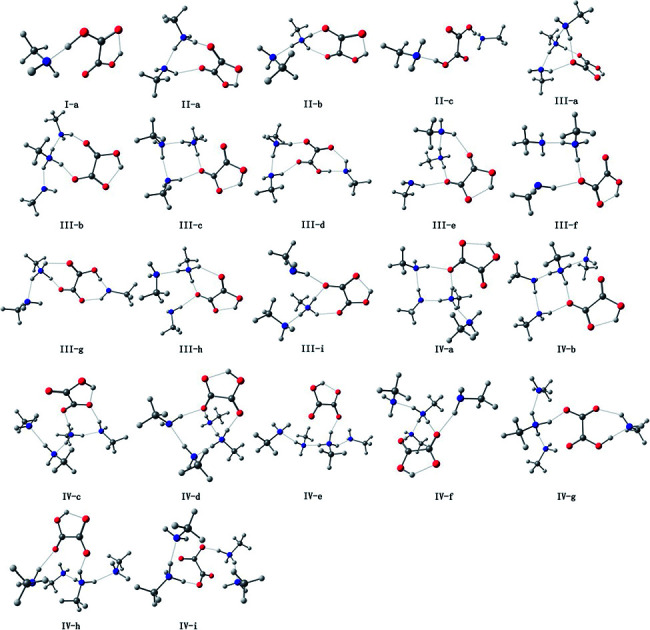

## 1. Introduction

Atmospheric aerosols, which are known to play a critical role in the global atmospheric system, are responsible for weather, climate, and human health.^1–4^ Primary aerosols are released into the air directly, and secondary aerosols are formed *via* various physical and chemical processes.^5,6^ New particle formation (NPF) is related to aerosol production and has been frequently observed in various environments, such as urban, forested, and remote continental areas.^7^ The formation of new particles is divided into two processes:^8–11^ a nucleation process to form a subcritical nucleus and a growth process in which the crucial clusters grow rapidly in size.^10,12^ Thus far, interest in NPF has constantly increased, and a large amount of theoretical and experimental work has been carried out.^13–27^ However, the accurate nucleation mechanisms and the species that participate in NPF are still uncertain.^2,10,28–30^

It has been proved that the concentration of sulfuric acid (SA) vapor can influence nucleation rates observably, according to various field and lab measurements.^10,31,32^ However, it is difficult for the binary H_2_SO_4_/H_2_O nucleation (BHN) mechanism to explain the measured nucleation rates in the lower atmosphere.^33–36^ Recently, interest in organic species in atmospheric nucleation has increased.^28,37–40^ Research into organic acids involved in nucleation, both pioneering laboratory experiments by Zhang *et al.* and theoretical studies by Yu *et al.*, has pointed out that organic acids have the ability to accelerate both nucleation and the subsequent growth.^39,41^

Most research has focused on the nucleation of organic with inorganic species, whereas few studies have been carried out on the nucleation of organic acids with organic species. Dicarboxylic acids, which are representatives of familiar organic acids in the atmosphere, are frequently observed at significant concentrations and are expected to participate in nucleation owing to their relatively low vapor pressures.^42–45^ Oxalic acid, as the most common dicarboxylic acid, is the major constituent of ultrafine and fine aerosol particles.^46–48^ Martinelango *et al.*^47^ indicated that the gas-phase concentrations of oxalic acid are high enough for it to play an important role in nucleation. Moreover, according to field measurements, a strong correlation exists between oxalic acid concentrations and cloud condensation nuclei (CCN), which implies that oxalic acid may accelerate the activation of CCN.^49^ Recently, it has been found that oxalic acid is effective in binding with ammonia, which indicates that oxalic acid and ammonia may form thermodynamically stable clusters.^50^ In addition, methylamine is one of the structurally simplest and lowest-molecular-weight organic amines in the atmosphere, and its estimated global emissions total 83 ± 26 Gg N a^−1^ (10^9^ g per annum).^51^ Concentrations of methylamine are less than those of ammonia in the atmosphere; however, they have increased strikingly in specific situations such as animal husbandry, fish processing, industry, automobiles, sewage treatment, biomass burning, bacterial culture, and the ocean.^52^ Methylamine is among the neglected species in atmospheric nucleation. Nucleation studies that involve methylamine are few. Thus, it is of significance and interest to investigate clusters composed of oxalic acid and methylamine molecules.

In this work, the structure and stability of (H_2_C_2_O_4_)(CH_3_NH_2_)_*n*_ (*n* = 1–4) in the aerosol phase have been thoroughly studied. The basin-hopping (BH) method,^53–55^ coupled with a density functional theory (DFT) method, was used to search for low-lying structures for each cluster size. The thermodynamic properties were also evaluated. According to the results, the temperature in the formation of (H_2_C_2_O_4_)(CH_3_NH_2_)_*n*_ (*n* = 1–4) was studied, and the nucleation mechanism of oxalic acid with methylamine was researched in general terms. In addition, Rayleigh scattering properties are among the most significant factors for global climate change.^56–59^ However, studies of the scattering properties of molecules are lacking, and no relevant references have been found for the (H_2_C_2_O_4_)(CH_3_NH_2_)_*n*_ (*n* = 1–4) system. Therefore, it is meaningful to investigate the Rayleigh scattering properties of these clusters.

## 2. Theoretical methods

The initial geometries of the monomers and (C_2_H_2_O_4_)(CH_3_NH_2_)_*n*_ (*n* = 1–4) clusters were determined using the BH global optimization technique in combination with DFT. We chose the generalized gradient approximation in the Perdew–Burke–Ernzerhof (PBE) functional and the double numerical plus d-functions (DND) basis set for the structural optimization of these systems, which was implemented in DMol.^3,60,61^ This method has been validated in our previous studies.^62–67^

The BH method included two procedures. Firstly, new configurations were generated *via* the random displacement of atoms; then, these configurations were optimized to the local minima. Secondly, the optimized local energy minima were used as criteria to accept or reject the initially generated structure spaces using the Boltzmann weighting at a finite temperature. To ensure that there were no imaginary frequencies, the frequency calculation for each stationary point was included. The convergence criteria were the default settings in the Gaussian 09 suite of programs.^68^

Single-point energy calculations were performed at the DF-LMP2-F12/VDZ-F12 (second-order Møller–Plesset perturbation theory-explicitly correlated methods with density fitting) level of theory on the basis of the optimized geometries at the PW91PW91/6-311++G(3df,3pd) level of theory using Molpro 2010.1.^69^ The zero-point corrected energies [*E*(0 K)], atmospheric-temperature enthalpies [*H*(*T*)] and Gibbs free energies [*G*(*T*)] were calculated at the PW91PW91/6-311++G(3df,3pd) level of theory. The PW91PW91 functional was chosen to be the specific DFT method used in this study owing to its fine performance in predictions of structural characteristics and the thermodynamics of cluster formation and satisfactory similarity in comparison with experimental results.^7,42,70,71^

Tables 1 and 2 present the benchmark work carried out for the (C_2_H_2_O_4_)(CH_3_NH_2_)_*n*_ (*n* = 1–4) cluster system. The binding energies, thermal contributions to the free energies and resulting Gibbs free energies were calculated using three functionals (ωB97X-D, PW91 and M06-2X) with the same 6-311++G(3df,3pd) basis set. In Table 1, the Gibbs free energy calculated by PW91 is similar to those calculated by M06-2X and ωB97X-D. On comparing the results of the PW91PW91 functional and the DF-LMP2-F12/VDZ-F12 method in Table 2, the difference between Δ*E*_PW91_ and Δ*E*_CCSD(T)_ is larger than that between Δ*E*_DF_ and Δ*E*_CCSD(T)_. It is generally seen that both the PW91PW91 functional and the DF-LMP2-F12/VDZ-F12 method overestimated the binding energies, but the DF-LMP2-F12/VDZ-F12 method was more accurate than the PW91PW91 functional. DFT methods were used to calculate Δ*E* because of their lower memory consumption. Moreover, the accuracy of electronic energies could be improved by the PW91PW91/6-311++G(3df,3pd) thermodynamic corrections combined with the DF-MP2-F12 single-point energies. Thus, the DF-LMP2-F12/VDZ-F12//PW91PW91/6-311++G(3df,3pd) method used in this study could be viewed as a compromise between accuracy and computational efficiency. A higher level of theory may change the exact order of these clusters in terms of energy, but we believe that the current paper would be a starting point for additional refinements at higher levels of theory.

**Table 1:** Binding energies, thermal contributions to the free energies and resulting Gibbs free energies calculated for the (C_2_H_2_O_4_)(CH_3_NH_2_) complex using all three functionals (ωB97X-D, PW91 and M06-2X) with the 6-311++G(3df,3pd) basis set

Method	Δ*E* (kcal mol^−1^)	Δ*G*_therm_ (kcal mol^−1^)	Δ*G* (kcal mol^−1^)
ωB97X-D	−15.94	11.88	−4.06
M06-2X	−16.41	11.75	−4.66
PW91PW91	−16.08	11.27	−4.81

**Table 2:** Binding energies calculated by PW91, DF-LMP2 and CCSD(T)-F12 methods for three clusters

	Δ*E*_PW91_	Δ*E*_DF_	Δ*E*_CCSD(T)_	ΔΔ*E*_CCSD(T)/PW91_	ΔΔ*E*_CCSD(T)/DF_
I-a	−16.08	−15.34	−12.66	3.42	2.68
II-a	−27.04	−26.78	−23.68	3.36	3.10
II-b	−26.53	−25.84	−22.85	3.68	2.99

According to the global minima of (C_2_H_2_O_4_)(CH_3_NH_2_)_*n*_ (*n* = 1–4) determined in this study, optical properties, such as the depolarization ratios and Rayleigh scattering intensities for natural light, of the pre-nucleation clusters were then calculated. We calculated the anisotropic polarizabilities and mean isotropic polarizabilities of the clusters at the CAM-B3LYP/aug-cc-pVDZ level of theory. The CAM-B3LYP/aug-cc-pVDZ level of theory was a good compromise between efficiency and accuracy. The mean isotropic polarizability *ā*, anisotropic polarizability Δ*α*, Rayleigh scattering intensity of natural light *R*_n_ and depolarization ratio *σ*_n_ are defined as follows:^72^1
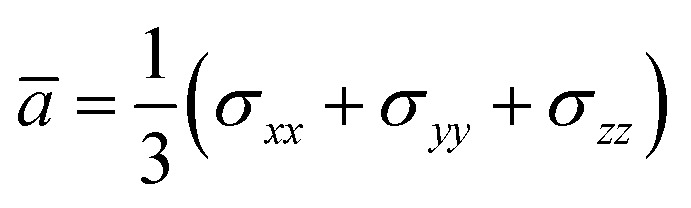
2
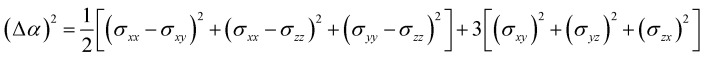
3
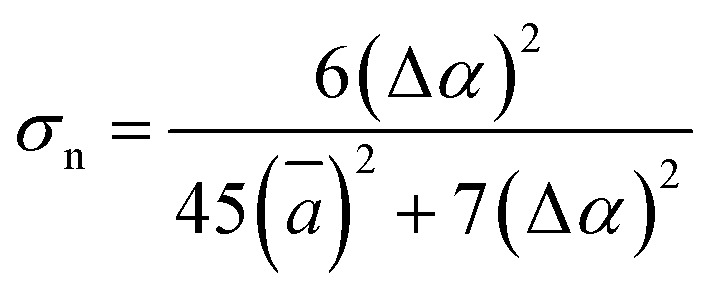

4 *R*_n_ = 45(*ā*)^2^ + 13(Δ*α*)^2^

## 3. Results and discussion

### 3.1 Structures

The oxalic acid monomer C_2_H_2_O_4_ contains two carboxyl groups. The geometry of oxalic acid is a planar *trans* conformation (*C*_2h_ point group) with intramolecular hydrogen bonds.^7,44^ In this section, the structures corresponding to the global and some specific local minima of (C_2_H_2_O_4_)(CH_3_NH_2_)_*n*_ (*n* = 1–4) at the PW91PW91/6-311++G(3df,3pd) level of theory are discussed, and *m*–*n* notation is used to define the conformations. In this notation, *m* (*m* = I, II, III, and IV) represents the number of methylamine molecules, and *n* (*n* = *a*–*f*), which is ordered according to increasing electronic energy at 0 K for each cluster size, is used to distinguish different isomers with the same value of *m*.

**Fig. 1:**
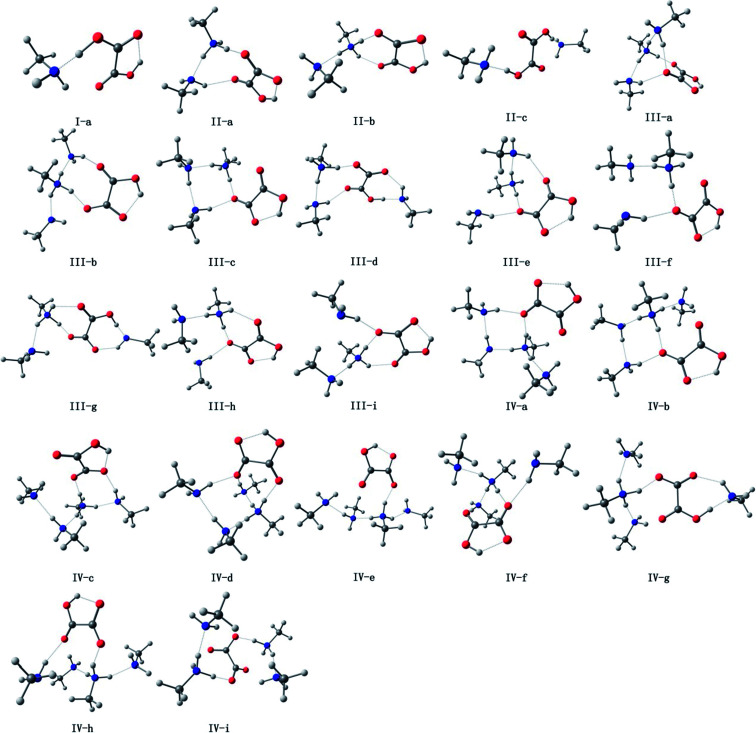
Optimized geometries of (H_2_C_2_O_4_)(CH_3_NH_2_)_*n*_ (*n* = 1–4) at the PW91PW91/6-311++G(3df,3pd) level of theory (red for oxygen, white for hydrogen, gray for carbon and blue for nitrogen).

**Fig. 2:**
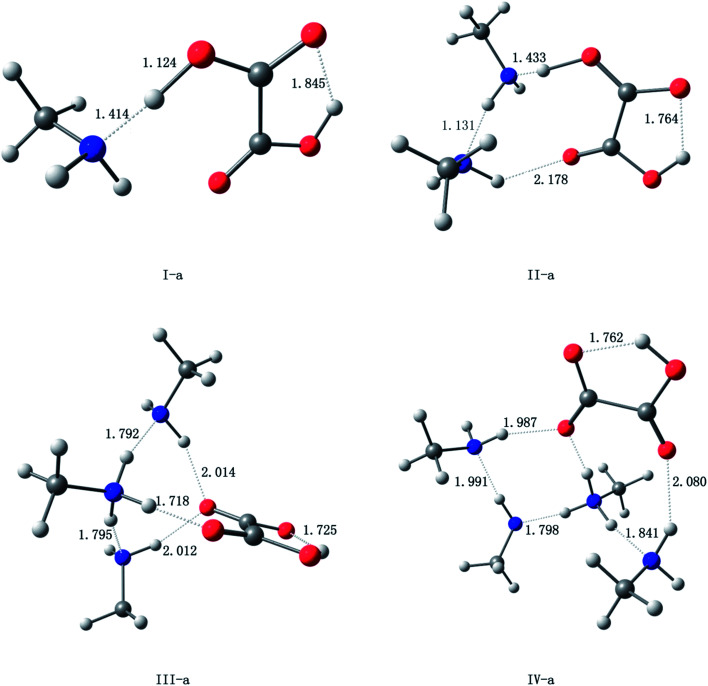
Lowest-energy structures of the (H_2_C_2_O_4_)(CH_3_NH_2_)_*n*_ (*n* = 1–4) clusters at the PW91PW91/6-311++G(3df,3pd) level of theory ordered according to the state of association (I, II, III, IV). The intramolecular and intermolecular interaction distances are given.

The optimized structures corresponding to the global and local minima are shown in Fig. 1. The intramolecular and intermolecular interaction distances for the global minimum structures are displayed in Fig. 2. As shown in Fig. 2, a strong N⋯H–O hydrogen bond with a length of 1.414 Å is formed in the (C_2_H_2_O_4_)(CH_3_NH_2_) cluster. It is observed that the global minimum structure for the (C_2_H_2_O_4_)(CH_3_NH_2_)_2_ cluster is II-a, which possesses a ring configuration. The length of the strong hydrogen bond increases from 1.414 Å to 1.433 Å. We can see that the more molecules participate in the ring-like structure, the more stable is the cluster. Besides, structures with two methylamine molecules on the same side of the oxalic acid molecule are more stable than those in which they are on opposite sides. For (C_2_H_2_O_4_)(CH_3_NH_2_)_3_, we located nine low-lying isomers. Isomer III-a has the lowest electronic energy. In this configuration, proton transfer occurred in which a methylamine molecule acted as an acceptor while the C_2_H_2_O_4_ molecule acted as a donor to form an HC_2_O_4_^−^/CH_3_NH_3_^+^ ion pair. The number of structures increases rapidly for the clusters with three methylamine molecules. Moreover, a dramatic structural change occurs in these clusters. The most stable structure (III-a) possesses a cage-like configuration, followed by ring-like structures. Among the structures with three methylamine molecules on the same side of the oxalic acid molecule, except for III-e, the more atoms participate in the ring-like structure, the more stable is the cluster, which is similar to the case of (C_2_H_2_O_4_)(CH_3_NH_2_)_2_. For the structures with three methylamine molecules on opposite sides of the oxalic acid molecule, the same conclusion applies. For isomer III-e, the hydrogen bond that connects the oxalic acid molecule and the methylamine molecule that does not participate in the ring is weak, and its length is 2.1 Å, which is longer than all the other hydrogen bonds. This may be the reason why III-e is less stable than III-a, b, c, and d. For the (C_2_H_2_O_4_)(CH_3_NH_2_)_4_ clusters, the diversity of structures increases. Two rings are formed for the most stable cluster, and the isomer that possesses a ring configuration is more stable than the isomer without the ring configuration. As shown in Fig. 2, a strong N⋯H–O hydrogen bond with a length of 1.798 Å is formed in the (C_2_H_2_O_4_)(CH_3_NH_2_)_4_ cluster IV-a, while a weaker O⋯H hydrogen bond with a length of 2.080 Å is formed in this (C_2_H_2_O_4_)(CH_3_NH_2_)_4_ cluster. There is also an interesting phenomenon, namely, the difference between the strong and weak bonds in (C_2_H_2_O_4_)(CH_3_NH_2_)_4_ is less than that in (C_2_H_2_O_4_)(CH_3_NH_2_)_*n*_ (*n* = 1–3).

### 3.2 Atmospheric relevance

Relevant calculations revealed that (C_2_H_2_O_4_)(CH_3_NH_2_)_*n*_ (*n* = 1–4) clusters have a noteworthy concentration in the atmosphere and could be participating in new particle formation. It is of significance to determine the actual concentration of clusters of oxalic acid with methylamine and investigate their effects under given realistic atmospheric conditions. A reference for the possible existence of (H_2_C_2_O_4_)(CH_3_NH_2_)_*n*_ (*n* = 1–4) clusters could be provided to estimate the actual concentration.^69^ The relative population fraction (RPF) is defined as follows:

5 RPF = [H_2_C_2_O_4_·*i*CH_3_NH_2_]/[H_2_C_2_O_4_]

With reference to the following equation:6
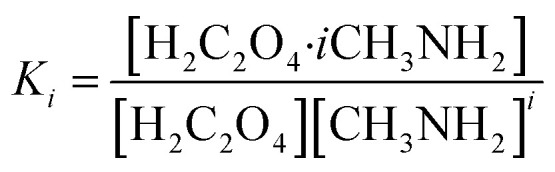
the relative population fraction (RPF) is then defined as follows:7
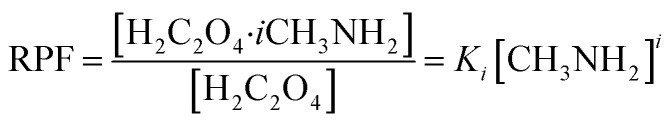
where *i* is the number of CH_3_NH_2_ molecules in each cluster and [X] represents the concentration of *X*. The total concentrations of oxalic acid and methylamine were selected as 5 × 10^11^ cm^−3^ and 1 × 10^7^ cm^−3^, respectively.^69,73^

The concentrations of all the clusters were calculated, and the results are listed in Table 3. It is predicted that the concentration of (H_2_C_2_O_4_)(CH_3_NH_2_) clusters in the atmosphere is 2.706 × 10^3^ molecules per cm^3^. These results can only be used as simple approximations, because the atmosphere is much more complex. However, we can perform an overall analysis from the data. RPF values are shown in Table 3 for (H_2_C_2_O_4_)(CH_3_NH_2_)_*n*_ (*n* = 1–4) at a temperature of 298.15 K. In addition, as the number of binding methylamine molecules increases, the concentrations of the clusters decrease rapidly, which could be because the concentration of methylamine is lower than that of oxalic acid in the atmosphere. We also found that the concentration of (H_2_C_2_O_4_)(CH_3_NH_2_)_4_ clusters is low at typical atmospheric temperatures in the boundary layer.

**Table 3:** Gibbs free energy^a^ values in kcal mol^−1^, relative bound percentages^b^ (RPF × 100%) and estimated concentrations of stable clusters of (H_2_C_2_O_4_)(CH_3_NH_2_)_*n*_ (*n* = 1–4)

*n*	Isomer	Δ*G* (kcal mol^−1^)	RPF × 100%	Molecules per cm^3^
1	I-a	−7.52	5.41 × 10^−7^	2705.80
2	II-a	−7.10	4.42 × 10^−19^	2.21 × 10^−11^
	II-b	−6.92	3.26 × 10^−19^	1.63 × 10^−11^
	II-c	−6.92	3.27 × 10^−19^	1.64 × 10^−11^
3	III-a	−7.76	2.25 × 10^−31^	1.13 × 10^−24^
	III-b	−7.97	3.19 × 10^−31^	1.60 × 10^−24^
	III-c	−6.54	2.86 × 10^−32^	1.43 × 10^−25^
	III-d	−5.50	4.98 × 10^−33^	2.49 × 10^−26^
	III-e	−4.77	1.44 × 10^−33^	7.22 × 10^−27^
	III-f	−3.28	1.16 × 10^−34^	5.82 × 10^−28^
	III-g	−4.38	7.44 × 10^−34^	3.72 × 10^−27^
	III-h	−4.17	5.24 × 10^−34^	2.62 × 10^−27^
	III-i	−4.56	1.01 × 10^−34^	5.03 × 10^−27^
4	IV-a	−7.91	4.86 × 10^−44^	2.43 × 10^−38^
	IV-b	−8.58	1.49 × 10^−43^	7.43 × 10^−38^
	IV-c	−6.24	2.88 × 10^−45^	1.44 × 10^−39^
	IV-d	−6.07	2.14 × 10^−45^	1.07 × 10^−39^
	IV-e	−6.13	2.39 × 10^−45^	1.19 × 10^−39^
	IV-f	−5.43	7.27 × 10^−46^	3.64 × 10^−40^
	IV-g	−6.18	2.61 × 10^−45^	1.31 × 10^−39^
	IV-h	−5.42	7.13 × 10^−46^	3.57 × 10^−40^
	IV-i	−4.48	1.47 × 10^−46^	7.34 × 10^−41^
	IV-j	−4.48	1.47 × 10^−46^	7.34 × 10^−41^
	IV-k	−1.33	7.13 × 10^−49^	3.57 × 10^−43^

a PW91PW91/6-311++G(3df,3pd) results.

b With respect to the corresponding clusters.

Concentrations of methylamine are less than those of ammonia in the atmosphere; however, they have increased strikingly in specific situations such as animal husbandry, fish processing, industry, automobiles, sewage treatment, biomass burning, bacterial culture, and the ocean,^52^ which will cause an increase in the concentration of (H_2_C_2_O_4_)(CH_3_NH_2_)_4_ clusters.

### 3.3 Temperature dependence of cluster formation

Previous studies^1,72^ showed that the energy differences between the global minimum and other local minima become smaller when cluster systems grow larger and their configurations grow more complex. In addition, researchers^72,73–75^ also found that the thermodynamic properties and variations in the population order of the isomers could be affected by differences in temperature, which revealed that the temperature dependence of cluster formation is a significant factor for understanding the nucleation mechanism. However, it is difficult to perform the relevant experiments, *i.e.*, the formation of clusters of oxalic acid with methylamine, at low temperatures owing to wall losses. Fortunately, relevant information could be obtained by quantum chemistry calculations. In this section, we calculated the relative populations of structures for the formation of clusters of H_2_C_2_O_4_ with CH_3_NH_2_ at temperatures ranging between 100 and 400 K. The equation used to calculate the relative populations at different temperatures is as follows:8
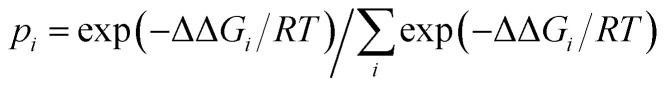
where *p*_*i*_ is the relative population of the *i*th isomer, ΔΔ*G*_*i*_ is the difference in Gibbs free energy between the *i*th isomer and the most stable isomer, *R* is the ideal gas constant, and *T* is the temperature.^76^

**Fig. 3:**
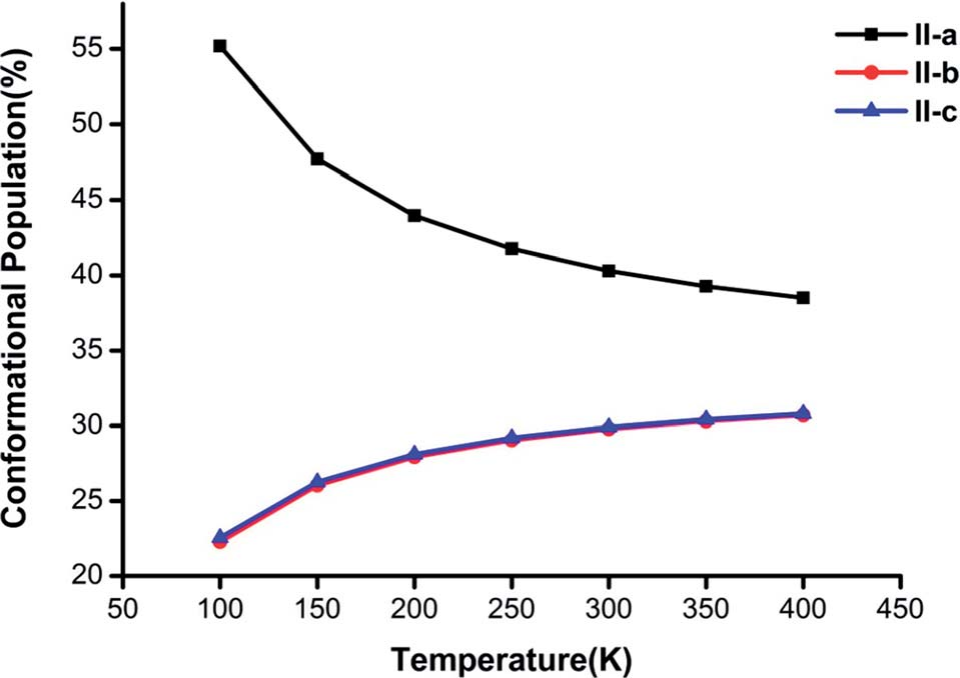
Changes in the conformational populations of the low-energy isomers of (H_2_C_2_O_4_)(CH_3_NH_2_)_2_ as a function of temperature.

**Fig. 4:**
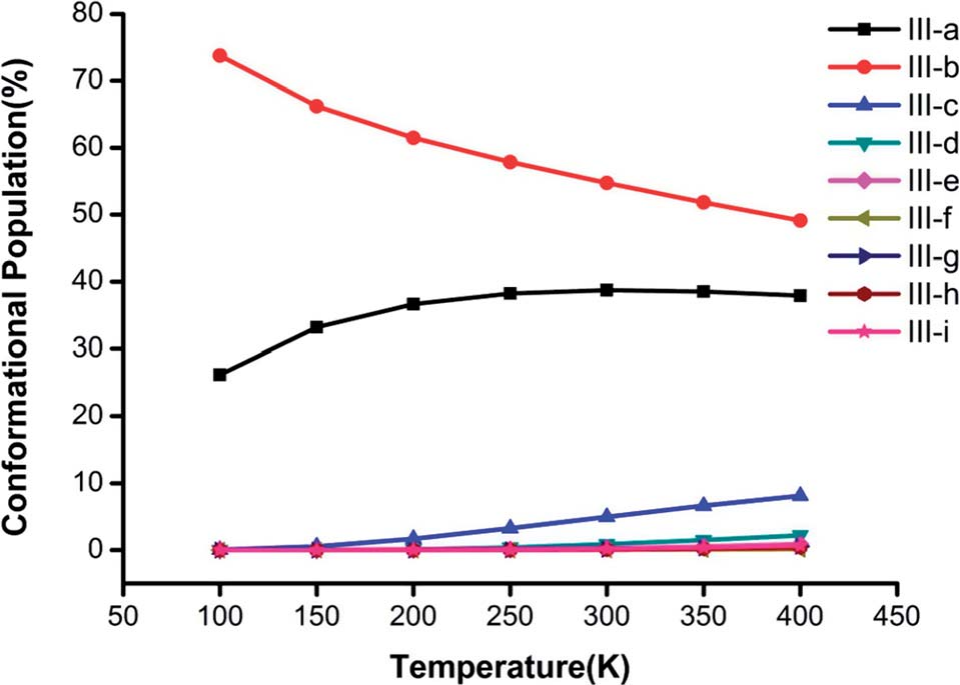
Changes in the conformational populations of the low-energy isomers of (H_2_C_2_O_4_)(CH_3_NH_2_)_3_ as a function of temperature.

**Fig. 5:**
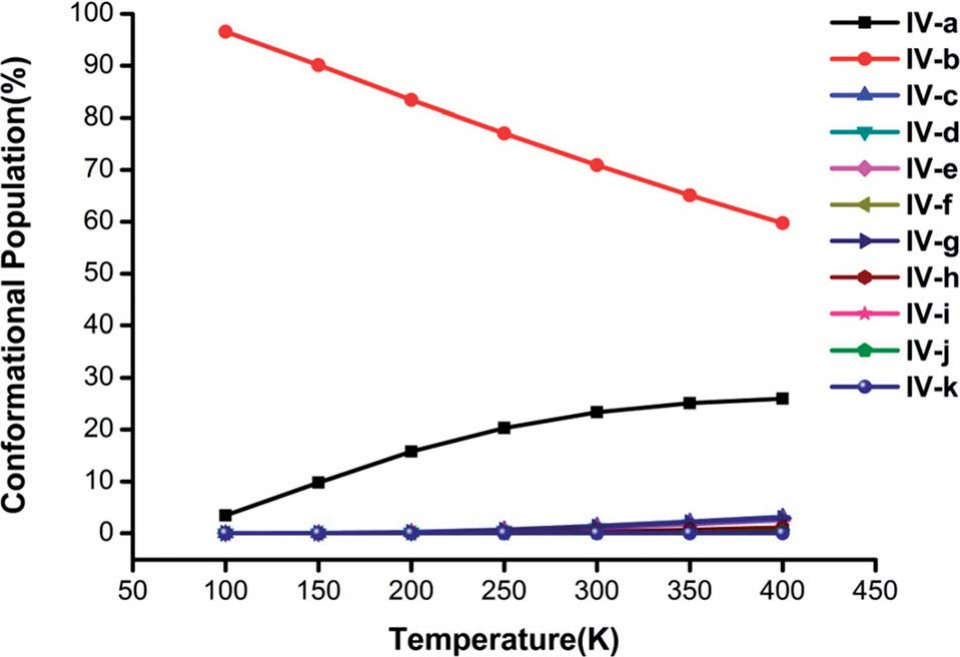
Changes in the conformational populations of the low-energy isomers of (H_2_C_2_O_4_)(CH_3_NH_2_)_4_ as a function of temperature.

Fig. 3–5 show the temperature dependence of the conformational populations for (H_2_C_2_O_4_)(CH_3_NH_2_)_2_, (H_2_C_2_O_4_)(CH_3_NH_2_)_3_, and (H_2_C_2_O_4_)(CH_3_NH_2_)_4_, respectively. It is observed that the relative populations of the most abundant isomers decrease rapidly while those of the other isomers increase with an increase in temperature.

For *n* = 2 in Fig. 3, the global minimum structure II-a accounts for the largest proportion, but its proportion undergoes a downward trend from 100 K to 400 K. The proportion of II-b is similar to that of II-c. The difference between II-b and II-c reaches a maximum of 0.28% at a temperature of 100 K and decreases with an increase in temperature to a minimum at *T* = 400 K.

For *n* = 3 in Fig. 4, the most stable isomer III-a is predominant in comparison with the other isomers, and its proportion exceeds 50% when the temperature is lower than 400 K. It is obvious that the proportions of the other isomers display a rising trend from 100 K to 400 K, and the proportion of structure III-c has the fastest rate of increase. The most stable structures, namely, III-a, III-b and III-c, have a combined proportion of greater than 95%, which decreases with an increase in temperature.

For *n* = 4 in Fig. 5, the percentage of isomer IV-b is the highest and is much greater than those of the other isomers, and its proportion exceeds 60% when the temperature is lower than 400 K. An interesting phenomenon is found, namely, that the conformational population could be thought of as a linear function of temperature. The combined proportion of structures IV-a and IV-b exceeds 95% and decreases with an increase in temperature, which is similar to the case of (H_2_C_2_O_4_)(CH_3_NH_2_)_3_ clusters in Fig. 4.

In general, it could be deduced from the Gibbs free energies of the (H_2_C_2_O_4_)(CH_3_NH_2_)_*n*_ (*n* = 2–4) clusters at various temperatures that the stability order of the isomers would be affected by temperature. As the temperature increases, the Δ*G* values indicate that the proportion of the global minimum structure decreases, whereas those of other local minimum structures increase. Although no H_2_O molecules were included in the calculations, significant information was still provided for an understanding of the formation processes of clusters of H_2_C_2_O_4_ with CH_3_NH_2_ in this work. Future work is needed to investigate the effects of H_2_O.

### 3.4 Thermodynamics of cluster formation

Thermodynamics analyses play an important role in assessing the probability and possibility of cluster formation. The strength of the intermolecular interactions and the spontaneity of cluster formation are investigated *via* changes in the Gibbs free energy. The binding energies, enthalpies, and Gibbs free energies (Δ*G*) of cluster formation for the (C_2_H_2_O_4_)(CH_3_NH_2_)_*n*_ (*n* = 2–4) clusters are shown in Table 4. The thermodynamic parameters were determined from the single-point electronic energies calculated by the DF-MP2-F12 method and thermal corrections performed using the PW91PW91 functional, as described in the Theoretical methods section. In Table 4, it can be seen that the formation of the heterodimer (C_2_H_2_O_4_)(CH_3_NH_2_) is exothermic by 15.34 kcal mol^−1^, and the formation of the trimer (C_2_H_2_O_4_)(CH_3_NH_2_)_2_ is exothermic by 26.78 kcal mol^−1^. Their formation would release 31–37 kcal mol^−1^ for the (H_2_C_2_O_4_)(CH_3_NH_2_)_3_ clusters and 42–46 kcal mol^−1^ for the (H_2_C_2_O_4_)(CH_3_NH_2_)_4_ clusters, respectively. The results of the calculations of Gibbs free energies are as follows: −7.52 kcal mol^−1^ for the (H_2_C_2_O_4_)(CH_3_NH_2_) cluster, −7.10 to −6.92 kcal mol^−1^ for the (H_2_C_2_O_4_)(CH_3_NH_2_)_2_ clusters, −7.97 to −3.28 kcal mol^−1^ for the (H_2_C_2_O_4_)(CH_3_NH_2_)_3_ clusters, and −8.58 to −1.33 kcal mol^−1^ for the (H_2_C_2_O_4_)(CH_3_NH_2_)_4_ clusters, all at room temperature, respectively. Obviously, the thermodynamics analysis revealed that it is favorable for an oxalic acid molecule to associate with up to four methylamine molecules. It can be seen from the Gibbs free energy that methylamine easily binds to oxalic acid in clusters. The Gibbs free energy decreases as the number of methylamine molecules increases, which indicates that clusters become more stable as the number of methylamine molecules increases, and larger clusters are more favorable in general.

**Table 4:** Relative single-point energies Δ*E*_rel_, ZPE-corrected binding energies (Δ*E*_0_), intermolecular enthalpies (Δ*H*), and changes in Boltzmann-averaged Gibbs free energies (Δ*G*) of (H_2_C_2_O_4_)(CH_3_NH_2_)_*n*_ (*n* = 1–4) (in kcal mol^−1^) based on PW91PW91/6-311++G(3df,3pd) calculations

*n*	Isomer	Δ*E*_rel_ (kcal mol^−1^)	Δ*E*_0_ (kcal mol^−1^)	Δ*H* (kcal mol^−1^)	Δ*G* (kcal mol^−1^)
1	I-a	0	−15.34	−17.03	−7.52
2	II-a	0	−26.78	−26.92	−7.10
	II-b	0.93	−25.84	−25.82	−6.92
	II-c	0.93	−25.85	−25.83	−6.92
3	III-a	0	−37.49	−37.51	−7.76
	III-b	0.71	−36.78	−36.57	−7.97
	III-c	1.65	−35.84	−35.81	−6.54
	III-d	2.06	−35.43	−35.56	−5.50
	III-e	4.00	−33.49	−33.21	−4.77
	III-f	4.78	−32.71	−33.21	−3.28
	III-g	4.38	−33.12	−32.98	−4.38
	III-h	5.24	−32.25	−31.88	−4.17
	III-i	5.25	−32.25	−31.86	−4.56
4	IV-a	0	−46.4	−46.68	−7.91
	IV-b	0.12	−46.82	−46.49	−8.58
	IV-c	0.83	−46.10	−45.93	−6.24
	IV-d	1.37	−45.57	−45.37	−6.07
	IV-e	2.29	−44.65	−44.24	−6.13
	IV-f	2.62	−44.31	−43.81	−5.43
	IV-g	2.33	−44.61	−44.30	−6.18
	IV-h	3.16	−43.78	−43.25	−5.42
	IV-i	3.64	−43.30	−42.83	−4.48
	IV-j	3.64	−43.30	−42.83	−4.48
	IV-k	4.42	−42.52	−42.80	−1.33

In our previous study concerning clusters of oxalic acid and ammonia molecules, the Gibbs free energies of the clusters ranged from −6.30 kcal mol^−1^ for (H_2_C_2_O_4_)(NH_3_) to 9.13 kcal mol^−1^ for the formation of (H_2_C_2_O_4_)(NH_3_)_*n*_ (*n* = 1–6).^77^ In comparison with ammonia, methylamine is more readily bound to oxalic acid, which promotes nucleation or new particle formation.

Recent studies^78,79^ showed that ammonia and alkylamines could promote the formation of sulfate aerosols. Shields *et al.* focused on a ternary system of sulfuric acid, methylamine, and up to six water molecules to evaluate its implications for aerosol formation.^78^ More recently, they discussed how ammonia and alkylamines act together and separately in the formation of sulfate aerosols.^79^ The discussions in both papers are instructive and give us a new direction for future research.

**Fig. 6:**
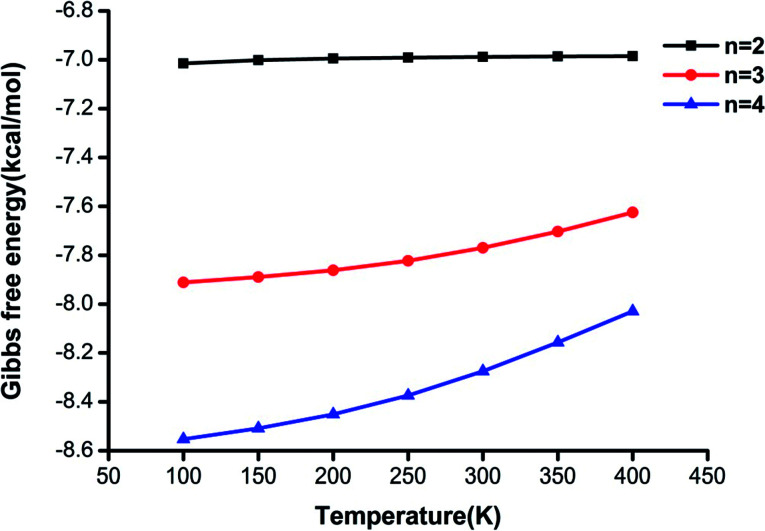
Changes in the Gibbs free energy (in kcal mol^−1^) of the global minimum structures of the (H_2_C_2_O_4_)(CH_3_NH_2_)_*n*_ (*n* = 2–4) clusters as a function of temperature at the PW91PW91/6-311++G(3df,3pd) level of theory.

In addition, Fig. 6 shows stepwise changes in the Boltzmann-averaged free energy of binding, which is calculated by the following equation:9
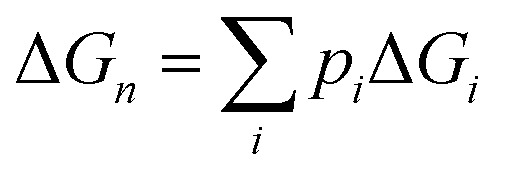


It is illustrated that the binding strength decreases from 100 K to 400 K for each cluster size, which means that the higher is the temperature, the less favorable is the formation of the clusters. The changes in Gibbs free energy for the (H_2_C_2_O_4_)(CH_3_NH_2_)_2_ cluster are less than those for the other clusters, and the difference in Gibbs free energy between 100 K and 400 K is only 0.03 kcal mol^−1^, which shows that this structure is less affected by temperature. The results also reveal that the clusters formed from oxalic acid and methylamine molecules could be stable in the atmosphere.

### 3.5 Optical properties

As we know, aerosols can scatter and absorb solar radiation, which has a great impact on climate.^1–4^ The light scattering intensity depends on the wavelength of the light and the particle size. Rayleigh scattering plays a leading role when the light wavelength is much greater than the particle diameter.^56^ However, the impact on climate of light scattering from atmospheric pre-nucleation clusters, except for the (H_2_C_2_O_4_)(CH_3_NH_2_)_*n*_ (*n* = 1–4) clusters, is still unclear. It is known that the Rayleigh scattering intensity is inversely proportional to the fourth power of the light wavelength. In this section, the polarizabilities and Rayleigh light scattering properties of the (H_2_C_2_O_4_)(CH_3_NH_2_)_*n*_ (*n* = 1–4) clusters are studied for the first time.

**Fig. 7:**
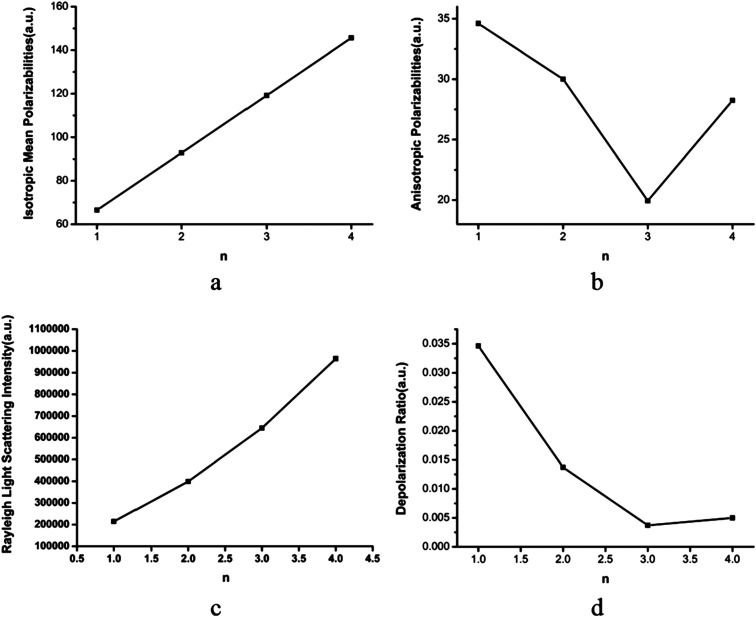
Rayleigh light scattering and polarizability properties of the clusters: (a) mean isotropic polarizability as a function of the number of methylamine molecules; (b) anisotropic polarizability as a function of the number of methylamine molecules; (c) Rayleigh light scattering intensity as a function of methylamine molecules; (d) depolarization ratio as a function of the number of methylamine molecules.

As shown in Fig. 7(a), it was found that the mean isotropic polarizability *ā* increases nearly linearly as the number of methylamine molecules increases, which is consistent with the study of clusters of oxalic acid with ammonia.^75^ From Fig. 7(b), we can see that the change in the anisotropic polarizability Δ*α* is non-monotonic with an increase in the number of methylamine molecules. It is seen that the anisotropic polarizability Δ*α* decreases from *n* = 1 to *n* = 3 and then increases from *n* = 3 to *n* = 4. The mean isotropic polarizability *ā* increases by approximately 80 au, whereas the change in the anisotropic polarizability Δ*α* is 5 au.

In Fig. 7(c), the Rayleigh scattering intensity of natural light *R*_n_ as a function of the number of methylamine molecules is observed to follow a second-order polynomial trend with a correlation coefficient of *ρ* = 0.99991. In Fig. 7(d), the depolarization ratio *σ*_n_ of the (H_2_C_2_O_4_)(CH_3_NH_2_)_*n*_ (*n* = 1–4) clusters can be seen as a function of the number of methylamine molecules in the clusters. Owing to the increase in the mean isotropic polarizability *ā* with the number of methylamine molecules and the relatively gentle variation in the anisotropic polarizability Δ*α*, the depolarization ratio *σ*_n_ exhibits a declining trend with an increase in the number of methylamine molecules.

Concerning the relationship between the number of hydrogen bonds and the polarizability of the cluster obtained from earlier studies, the calculated values of *ā* were fitted as a linear function of the cluster size *n*, *i.e.*, *n̄*_H_:

10 *ᾱ* = *a* + *b* × *n* + *c* × *n̄*_O–H_ + *d* × *n̄*_N–H_

where *n̄*_O–H_ = *n*_O–H_/*n* and *n̄*_N–H_ = *n*_N–H_/*n* represent the average numbers of O–H and N–H hydrogen bonds in the clusters, respectively. The fit was found to be excellent with a correlation coefficient of 0.9998, which reveals that both the O–H and the N–H hydrogen bonds in the (H_2_C_2_O_4_)(CH_3_NH_2_)_*n*_ (*n* = 1–4) system could contribute to the polarizability of the cluster.

## 4. Conclusions

In this study, the interactions of oxalic acid with methylamine and their hydrogen-bonded complexes were investigated *via* high-level DFT calculations. The global minimum and many local minima were determined for each cluster size. We also discussed the atmospheric relevance, temperature dependence and Rayleigh scattering properties. The results of this study lead to the following conclusions:

(a) Oxalic acid interacts strongly with methylamine, which means that strong hydrogen bonds are formed in the (C_2_H_2_O_4_)(CH_3_NH_2_)_*n*_ (*n* = 1–4) clusters. It is also predicted that the more atoms participate in a ring-like structure, the more stable is the cluster.

(b) From the thermodynamics and concentration data, it can be seen that there is a high probability that oxalic acid forms clusters with methylamine molecules in the atmosphere. The free energies at different temperatures show that the formation of (C_2_H_2_O_4_)(CH_3_NH_2_)_*n*_ (*n* = 1–4) clusters is more favorable under low-temperature conditions. As the temperature increases, the Δ*G* values indicate that the proportion of the global minimum structure decreases, whereas those of other local minimum structures increase. We also found that methylamine is more readily bound to oxalic acid than ammonia, which promotes nucleation or new particle formation. (C_2_H_2_O_4_)(CH_3_NH_2_) clusters have an evident concentration in the atmosphere, and these clusters could probably participate in new particle formation. However, as the number of binding methylamine molecules increases, the concentrations of the clusters decrease rapidly, which could be because the concentration of methylamine is lower than that of oxalic acid in the atmosphere.

(c) In this system, it can be indicated that the mean isotropic polarizability has an almost linear relationship with the number of methylamine molecules, whereas the Rayleigh scattering intensity follows a second-order polynomial trend with the number of methylamine molecules.

## Conflicts of interest

There are no conflicts to declare.

## Supplementary Material

RA-008-C7RA13670F-s001
