# Supplementary material for: Interaction of oxalic acid with methylamine and its atmospheric implications
Source: RSC Adv. 2018 Feb 14;8(13):7225–34. doi: 10.1039/c7ra13670f (PMC9078381; doi:10.1039/c7ra13670f)
Supplement: RA-008-C7RA13670F-s001 [file RA-008-C7RA13670F-s001.pdf]

Electronic Supplementary Material (ESI) for RSC Advances.
This journal is © The Royal Society of Chemistry 2018

Electronic Supporting Information for:

Interaction of Oxalic Acid with Methylamine and Its Atmospheric Implications

Yu Hong,[1,2] Yi-Rong Liu,[2] Hui Wen,[1] Shou-Kui Miao,[1,2] Teng Huang,[1] Xiu-Qiu Peng,[1,2] Shuai Jiang,[2] Ya-Juan Feng,[2] Wei Huang[1,2,3,*]

[1]*Laboratory of Atmospheric Physico-Chemistry, Anhui Institute of Optics & Fine Mechanics, Chinese Academy of Sciences, Hefei, Anhui 23 0031, China*

[2]*School of Information Science and Technology, University of Science and Technology of China, Hefei, Anhui 230026, China*

[3]*CAS Center for Excellent in Urban Atmospheric Environment, Institute of Urban Environment, Chinese Academy of Sciences, Xiamen, Fujian 361021, China*

*E-mail: huangwei6@ustc.edu.cn

List of Contents

Table S1. PW91PW91/6-311++G(3df,3pd) optimized Cartesian coordinates and single point energies for $H_2C_2O_4$-CH_3NH_2 isomers.

Table S2. PW91PW91/6-311++G(3df,3pd) optimized Cartesian coordinates and single point energies for $H_2C_2O_4$- $(CH_3NH_2)_2$ isomers.

Table S3. PW91PW91/6-311++G(3df,3pd) optimized Cartesian coordinates and single point energies for $H_2C_2O_4$- $(CH_3NH_2)_3$ isomers.

Table S4. PW91PW91/6-311++G(3df,3pd) optimized Cartesian coordinates and single point energies for $H_2C_2O_4$- $(CH_3NH_2)_4$ isomers.

Table S1.

$H_2C_2O_4$- CH_3NH_2
15
I-a E[DF-MP2-F12] -473.6629682

C	-0.975404	0.813045	-0.031767
C	-1.113210	-0.748773	-0.006552
O	0.194342	1.335741	-0.215175
O	-2.021921	1.438305	0.131678
O	-0.210562	-1.549698	-0.143532
O	-2.383748	-1.089408	0.191935
H	1.068331	0.645684	-0.358266
H	-2.840943	-0.204720	0.260757
C	3.203963	0.004365	0.585565
H	4.045389	-0.696583	0.512729
H	3.593686	1.027248	0.591071
H	2.687114	-0.167607	1.534912
N	2.235639	-0.145700	-0.515518
H	1.800210	-1.072751	-0.512715
H	2.679768	-0.002727	-1.422593

Table S2.

$H_2C_2O_4$-(CH_3NH_2)$_2$
22
II-a E[DF-MP2-F12] -569.3872719

C	1.644113	0.704589	0.469208
C	1.541616	-0.479028	-0.562458
O	0.613734	1.417201	0.662131
O	2.768500	0.793480	0.998664
O	0.549634	-0.783336	-1.205920
O	2.700444	-1.124212	-0.633369
H	-0.580284	1.194418	-0.093896
H	3.246303	-0.590551	0.028444
C	-2.017107	-1.984161	1.255481
H	-1.224154	-1.381657	1.713233
H	-2.956513	-1.746124	1.767378
H	-1.785320	-3.044491	1.434792
N	-2.127307	-1.630117	-0.169966
H	-2.855759	-2.183159	-0.620675
H	-1.246067	-1.836788	-0.649966
C	-2.531281	2.142425	-0.195116
H	-2.700638	2.027391	0.879094
H	-2.114696	3.137519	-0.375436

H -3.486320 2.044982 -0.721992
N -1.571660 1.109097 -0.635004
H -1.359667 1.180168 -1.632198
H -1.926667 0.117412 -0.468730

22
II-b E[DF-MP2-F12] -569.3857189
C 1.777033 0.126134 -0.714363
C 1.558968 -0.195334 0.818388
O 2.993145 -0.258629 -1.081649
O 0.974122 0.640246 -1.478564
O 2.552441 -0.741767 1.337411
O 0.452242 0.107012 1.356023
H 3.337181 -0.629728 -0.205614
H -0.597961 0.732944 0.580042
C -1.903390 2.464669 0.368115
H -2.609018 2.887317 -0.354701
H -2.412032 2.323949 1.326428
H -1.074818 3.163987 0.512258
N -1.377628 1.168950 -0.100128
H -0.823327 1.243674 -0.967056
H -2.129943 0.442038 -0.227181
C -2.171153 -2.194735 -0.159409
H -1.560393 -2.032815 0.735344
H -2.614633 -3.199374 -0.107581
H -1.500780 -2.157262 -1.025034
N -3.166685 -1.107557 -0.272271
H -3.818753 -1.150750 0.511755
H -3.729685 -1.233023 -1.114023

22
II-c E[DF-MP2-F12] -569.3857158
C -1.776863 0.126302 -0.714358
C -1.558936 -0.195094 0.818469
O -2.992823 -0.258765 -1.081770
O -0.973920 0.640436 -1.478558
O -2.552440 -0.741657 1.337322
O -0.452265 0.107201 1.356237
H -3.336835 -0.629838 -0.205684
H 0.597961 0.733104 0.580432
C 1.903556 2.464711 0.367744
H 2.609031 2.887087 -0.355385
H 1.075007 3.164085 0.511762
H 2.412400 2.324404 1.326012

N	1.377702	1.168829	-0.099901
H	2.129958	0.441851	-0.226875
H	0.823210	1.243255	-0.966778
C	2.170584	-2.194837	-0.159610
H	1.559500	-2.032811	0.734908
H	1.500571	-2.157121	-1.025506
H	2.613728	-3.199621	-0.107659
N	3.166458	-1.107960	-0.271990
H	3.729677	-1.233365	-1.113590
H	3.818209	-1.151317	0.512276

Table S3.

$H_2C_2O_4$-($CH_3NH_2)_3$

29

III-a E[DF-MP2-F12] -665.1125584

C	1.707335	-0.002628	-0.638926
C	1.798868	0.000526	0.937321
O	0.561781	-0.002075	-1.153562
O	2.844076	-0.005351	-1.168104
O	0.855793	0.003325	1.722273
O	3.064749	-0.000312	1.321792
H	-0.732147	0.002527	1.097922
H	3.514554	-0.002789	0.409118
C	-0.937668	-3.415299	-0.560096
H	-1.775631	-4.006886	-0.172822
H	-0.389528	-4.030448	-1.289860
H	-0.260896	-3.207564	0.277046
N	-1.433747	-2.142201	-1.105076
H	-2.051096	-2.312490	-1.898605
H	-0.646135	-1.572131	-1.444061
C	-2.730906	0.004607	1.798712
H	-3.736837	0.004361	1.367299
H	-2.610940	-0.883798	2.425932
H	-2.610055	0.895060	2.422852
N	-1.720368	0.002305	0.724035
H	-1.784967	0.855177	0.086412
H	-1.785837	-0.852444	0.089065
C	-0.931069	3.414060	-0.564489
H	-0.380524	4.027567	-1.293824
H	-1.767915	4.007944	-0.178314
H	-0.255747	3.204792	0.273447
N	-1.429850	2.142085	-1.109624
H	-0.643263	1.569851	-1.447340
H	-2.045838	2.313664	-1.903922

29
III-b E[DF-MP2-F12] -665.1111002
C 1.864446 -1.295859 0.143684
C 0.509741 -2.071719 -0.046471
O 2.890932 -2.137749 0.064778
O 1.979035 -0.095403 0.329116
O 0.668506 -3.294149 -0.242672
O -0.556857 -1.396032 0.011031
H 2.400675 -3.005498 -0.095824
H -0.527444 0.159636 0.293447
C -0.939438 1.587132 1.805752
H 0.060830 1.521810 2.243515
H -1.311358 2.612659 1.896226
H -1.606689 0.911848 2.349411
N -0.871546 1.182113 0.387899
H -0.202495 1.796767 -0.156819
H -1.816561 1.126991 -0.063291
C 1.898264 3.768972 -0.430654
H 2.917345 3.949664 -0.805874
H 1.275214 4.628578 -0.704299
H 1.947295 3.728221 0.663504
N 1.285478 2.530280 -0.933540
H 1.843231 1.714938 -0.657027
H 1.263874 2.537565 -1.953389
C -4.434599 -0.338122 -0.154436
H -4.229773 -0.509636 0.908642
H -5.114140 0.519162 -0.228019
H -4.958098 -1.226199 -0.541584
N -3.173273 -0.033667 -0.846913
H -3.345821 0.091537 -1.844527
H -2.524114 -0.824884 -0.761489

29
III-c E[DF-MP2-F12] -665.1093834
C -1.815878 0.584872 -0.472037
C -2.347072 -0.391739 0.646857
O -0.759512 0.262604 -1.096329
O -2.518906 1.605315 -0.606717
O -1.821981 -1.429626 1.013148
O -3.473170 0.086852 1.168692
H 1.550499 -1.478945 -0.074010
H -3.576459 0.940165 0.643797
C 3.203799 -0.353564 1.736051

H	3.958701	0.411382	1.974848
H	2.263346	-0.054875	2.214295
H	3.521090	-1.299107	2.191992
N	2.976656	-0.534699	0.293237
H	3.846036	-0.812581	-0.163271
H	2.681962	0.368621	-0.138120
C	0.709200	-2.928146	-1.394160
H	-0.287781	-3.250914	-1.707601
H	1.244315	-2.537855	-2.264761
H	1.257046	-3.786096	-0.991780
N	0.592658	-1.862417	-0.379948
H	0.031730	-2.157600	0.429397
H	0.012987	-1.006601	-0.736858
C	1.518845	3.044973	0.068882
H	0.839993	3.834851	-0.289157
H	1.089830	2.634810	0.990970
H	2.478918	3.507598	0.328819
N	1.733724	1.952388	-0.892846
H	2.113886	2.330999	-1.760673
H	0.827820	1.529715	-1.134891

29

III-d E[DF-MP2-F12] -665.1068096

C	0.426019	-0.074783	0.755990
C	0.703518	-1.190194	-0.324357
O	1.433704	0.658459	1.172039
O	-0.714363	0.102273	1.182713
O	1.857921	-1.624809	-0.448399
O	-0.320811	-1.559214	-0.991233
H	2.396419	0.417826	0.794017
H	-1.615082	-1.141014	-0.726064
C	-2.311247	2.755317	-0.832274
H	-1.484015	2.281359	-1.373467
H	-3.098336	2.985770	-1.560068
H	-1.939578	3.703783	-0.414818
N	-2.823211	1.815785	0.176491
H	-3.576986	2.244086	0.712909
H	-2.075532	1.568525	0.835607
C	-3.269281	-1.776642	0.504012
H	-4.336135	-1.592614	0.670928
H	-3.111906	-2.834312	0.273389
H	-2.703011	-1.520978	1.403290
N	-2.747654	-0.943514	-0.601535
H	-2.884647	0.085588	-0.383243

H -3.217688 -1.161838 -1.482117
C 4.429025 1.014546 -0.690985
H 4.642431 1.997467 -0.255276
H 5.354374 0.624881 -1.140955
H 3.695280 1.154694 -1.492341
N 3.852411 0.130567 0.328839
H 4.511356 -0.047617 1.085470
H 3.552426 -0.768612 -0.069114

29
III-e E[DF-MP2-F12] -665.1053288
C 1.136904 -1.515046 -0.057830
C 2.280567 -0.452431 -0.250611
O -0.038430 -1.180585 -0.392120
O 1.541663 -2.588227 0.430362
O 2.137270 0.681846 -0.677598
O 3.446672 -0.957853 0.138750
H -0.328983 0.176623 -1.010430
H 3.159704 -1.880314 0.429411
C -3.849198 -1.771763 0.468105
H -4.836142 -1.371358 0.732268
H -3.828629 -1.895750 -0.621903
H -3.758244 -2.775881 0.916097
N -2.804167 -0.821287 0.865776
H -1.881785 -1.190515 0.611262
H -2.802996 -0.715128 1.880179
C -2.074487 1.189223 -1.759153
H -2.630399 0.857858 -0.873061
H -2.371626 2.203082 -2.046308
H -2.282594 0.502539 -2.585043
N -0.630081 1.147263 -1.429385
H -0.349586 1.879049 -0.702803
H -0.035007 1.309165 -2.244521
C -0.015656 2.482313 1.915826
H 0.665132 2.814875 2.713402
H -1.006649 2.909550 2.105367
H -0.107171 1.392033 1.977145
N 0.428410 2.851231 0.560777
H 1.348740 2.445218 0.367776
H 0.520924 3.863280 0.477819

29
III-f E[DF-MP2-F12] -665.1039141
C -2.728735 -0.459824 -0.346029

C -1.957512 0.839611 0.109871
O -3.976705 -0.148567 -0.681213
O -2.279460 -1.593210 -0.399203
O -2.657855 1.868343 0.052539
O -0.748491 0.721365 0.473672
H -3.971419 0.846277 -0.516693
H -0.047014 -0.608225 0.451183
C 0.825018 -2.073822 1.747914
H 1.232346 -3.088555 1.696931
H 1.589863 -1.393934 2.133639
H -0.034539 -2.063544 2.424068
N 0.404526 -1.610850 0.409672
H -0.384196 -2.156347 0.034352
H 1.210876 -1.596150 -0.278284
C 3.798583 -0.850160 -0.601053
H 4.648805 -0.529744 -1.221522
H 3.474938 -0.007796 0.025255
H 4.140472 -1.661362 0.052348
N 2.635857 -1.318088 -1.384973
H 2.904089 -2.083508 -2.004296
H 2.309396 -0.564532 -1.992112
C 2.392224 2.882243 -0.232270
H 1.890721 3.859027 -0.125156
H 3.476097 3.055916 -0.217917
H 2.137111 2.493759 -1.226672
N 2.030147 1.887211 0.783816
H 1.018084 1.720499 0.756367
H 2.233275 2.258569 1.711946

29
III-g E[DF-MP2-F12] -665.1033331
C -0.544757 -0.398218 0.288580
C -0.981782 0.919120 -0.451740
O 0.612114 -0.366497 0.837515
O -1.319325 -1.366735 0.263954
O -0.161378 1.609798 -1.055397
O -2.255278 1.246394 -0.419047
H 1.561281 0.656353 0.481554
H -2.897108 0.560270 0.067089
C 4.282544 -2.171662 -0.081356
H 4.404742 -1.849025 0.959107
H 4.116764 -3.260313 -0.078234
H 5.224895 -1.970551 -0.604145
N 3.197447 -1.398785 -0.703683

H 2.313014 -1.567228 -0.211103
H 3.051923 -1.709636 -1.664457
C 3.094486 2.213087 0.823177
H 2.388561 2.923509 1.263375
H 3.593489 1.668048 1.630171
H 3.844865 2.765075 0.247338
N 2.364133 1.261795 -0.029870
H 2.960742 0.504699 -0.442840
H 1.795440 1.710856 -0.762671
C -5.054623 -0.769403 -0.252203
H -4.659930 -0.943876 -1.259045
H -5.629884 -1.657233 0.050172
H -5.738025 0.085670 -0.305067
N -3.936117 -0.456723 0.646521
H -3.235547 -1.208131 0.663434
H -4.257731 -0.293726 1.599594

29
III-h E[DF-MP2-F12] -665.1030927
C 2.367651 -0.424757 -0.587763
C 1.726381 0.644085 0.381664
O 3.508417 0.043344 -1.081333
O 1.911796 -1.521298 -0.872026
O 2.396842 1.692215 0.458657
O 0.635808 0.358796 0.962206
H 3.543063 0.950693 -0.640998
H -0.088813 -0.934363 0.691145
C -1.568592 3.340269 -0.014316
H -0.840037 4.070305 0.376481
H -1.187537 2.985088 -0.980318
H -2.510415 3.868823 -0.209727
N -1.816423 2.188097 0.861230
H -2.167892 2.512230 1.762189
H -0.929925 1.708084 1.052807
C -0.583211 -2.819005 1.582891
H 0.416743 -2.923265 2.013165
H -1.251673 -2.399292 2.340182
H -0.952036 -3.804983 1.282895
N -0.527050 -1.906718 0.423021
H -1.482754 -1.765475 -0.017580
H 0.161755 -2.219166 -0.278765
C -2.962080 -0.121797 -1.494954
H -3.885303 0.187979 -2.006174
H -2.648556 0.678245 -0.810527

H -2.181472 -0.263426 -2.251580
N -3.114332 -1.370427 -0.714437
H -3.509689 -2.109336 -1.297164
H -3.784610 -1.216037 0.040098

29
III-i E[DF-MP2-F12] -665.1030557
C -2.720184 -0.498622 -0.190586
C -1.918546 0.858755 -0.098673
O -3.978946 -0.254719 -0.537943
O -2.284035 -1.620711 0.012971
O -2.614109 1.853066 -0.382440
O -0.696280 0.815304 0.235245
H -3.957052 0.750488 -0.622504
H -0.407593 -2.069452 0.452228
C 3.664312 -0.966848 -0.921225
H 4.449440 -0.915199 -1.690270
H 3.327191 0.053026 -0.687996
H 4.100984 -1.410345 -0.018453
N 2.487689 -1.763164 -1.332101
H 2.771279 -2.699003 -1.624252
H 2.055837 -1.330813 -2.150507
C 0.917163 -1.683062 2.032828
H 1.738367 -0.985167 2.219711
H 0.108006 -1.475258 2.738691
H 1.272825 -2.707294 2.183268
N 0.427190 -1.497319 0.652365
H 1.182837 -1.675979 -0.071794
H 0.007574 -0.493895 0.494775
C 2.491092 2.823785 0.791246
H 1.955593 3.762174 1.015586
H 2.411074 2.181848 1.677945
H 3.553926 3.064717 0.659833
N 1.994719 2.094458 -0.381081
H 2.044448 2.695770 -1.203612
H 1.002008 1.868990 -0.251148

Table S4.
$H_2C_2O_4$-($CH_3NH_2)_4$
36
IV-a E[DF-MP2-F12] -760.8345915
C -1.603488 1.541350 0.137303
C -2.706470 0.421439 0.089980
O -0.433300 1.193585 0.462374

O	-2.037898	2.671389	-0.171557
O	-2.534998	-0.761610	0.329994
O	-3.876966	0.946406	-0.264606
H	0.006562	-0.319065	0.817505
H	-3.615117	1.914253	-0.377588
C	0.131979	-1.625739	2.458574
H	-0.954565	-1.715155	2.546140
H	0.600979	-2.579707	2.720267
H	0.482192	-0.852311	3.148715
N	0.477577	-1.247760	1.073034
H	1.527605	-1.100453	0.953640
H	0.117211	-1.973316	0.393900
C	2.561008	2.816713	-1.201818
H	3.631360	3.008808	-1.348785
H	2.258258	2.061624	-1.937559
H	2.010858	3.743129	-1.433503
N	2.323929	2.294677	0.151163
H	1.317613	2.115293	0.279579
H	2.579036	3.002404	0.840380
C	3.830009	-1.121913	-0.539197
H	4.034023	-2.189479	-0.389878
H	3.129893	-1.034553	-1.379244
H	4.771204	-0.633765	-0.838296
N	3.213493	-0.547437	0.665280
H	3.025748	0.468683	0.520304
H	3.857961	-0.619815	1.453167
C	-0.508989	-2.711356	-2.200758
H	0.421226	-3.236561	-2.448360
H	-1.278870	-3.014255	-2.927112
H	-0.330352	-1.637380	-2.330857
N	-0.868089	-2.971818	-0.798783
H	-1.058703	-3.963439	-0.658285
H	-1.721493	-2.459686	-0.553131

36

IV-b E[DF-MP2-F12] -760.8341884

C	2.585786	-0.913496	0.376223
C	1.326789	-1.765000	-0.026937
O	3.685409	-1.659817	0.305751
O	2.579805	0.261311	0.702473
O	1.608598	-2.942972	-0.333246
O	0.202576	-1.188750	-0.006837
H	3.289940	-2.538601	0.006787
H	-0.026991	0.352489	0.408554

C	-0.356412	1.495754	2.137734
H	-1.020385	0.755473	2.593969
H	0.660773	1.335743	2.506483
H	-0.693844	2.499937	2.413771
N	-0.369502	1.335230	0.668705
H	-1.352660	1.431873	0.268981
H	0.295746	2.024518	0.221726
C	-4.163100	1.438105	0.463270
H	-4.021942	0.838974	1.370957
H	-4.234007	2.489170	0.769571
H	-5.129153	1.147227	0.020293
N	-3.011486	1.243133	-0.429571
H	-3.144205	1.782735	-1.285853
H	-2.947053	0.241660	-0.715197
C	-3.305316	-2.657157	-0.227693
H	-4.363097	-2.650239	-0.519675
H	-2.935329	-3.692563	-0.303785
H	-3.253852	-2.365907	0.828600
N	-2.553849	-1.685633	-1.033913
H	-2.577856	-1.964389	-2.015087
H	-1.562163	-1.698254	-0.754912
C	1.838722	2.910203	-1.929174
H	1.513965	1.935578	-2.312023
H	1.152416	3.668619	-2.324075
H	2.843211	3.112222	-2.332046
N	1.771546	2.896435	-0.460233
H	2.087019	3.789129	-0.081947
H	2.392589	2.171825	-0.085671

36

IV-c E[DF-MP2-F12] -760.8329922

C	-0.334203	1.499434	-0.663009
C	-1.533132	2.247016	0.014093
O	-0.582387	0.395483	-1.204793
O	0.769833	2.105936	-0.549329
O	-2.642868	1.787413	0.176005
O	-1.162179	3.465391	0.436027
H	0.590497	-0.697692	-1.150267
H	-0.194140	3.477802	0.198331
C	1.563615	-2.435676	-1.902014
H	0.633443	-2.967967	-2.124366
H	1.917991	-1.950611	-2.816844
H	2.315438	-3.158040	-1.567766
N	1.322811	-1.425415	-0.855916

H 0.912966 -1.860311 0.037169
H 2.191706 -0.851688 -0.638912
C 0.304906 -1.507489 2.609772
H 0.334440 -0.447827 2.328372
H -0.392626 -1.606226 3.456002
H 1.306316 -1.788477 2.959252
N -0.069529 -2.307623 1.432919
H -1.028046 -2.029389 1.120109
H -0.115600 -3.294938 1.687191
C -3.104316 -2.047215 -0.819101
H -3.214710 -3.124198 -0.635569
H -4.033107 -1.685003 -1.289217
H -2.294069 -1.904318 -1.543213
N -2.749425 -1.355705 0.427643
H -3.530237 -1.404471 1.082952
H -2.612802 -0.357592 0.226000
C 3.911086 0.740224 0.945769
H 3.159492 0.546292 1.720159
H 4.722992 0.015642 1.082190
H 4.317848 1.748490 1.120498
N 3.296403 0.564722 -0.377965
H 3.979997 0.758110 -1.109990
H 2.523468 1.239014 -0.505197

36
IV-d E[DF-MP2-F12] -760.8323118
C -0.316598 1.877211 0.141894
C -0.962074 1.727302 -1.286534
O -0.167361 0.829000 0.817838
O -0.020219 3.068278 0.401988
O -1.308771 0.678760 -1.817303
O -1.077667 2.912434 -1.867735
H -0.947003 -0.798237 -0.976273
H -0.677741 3.497081 -1.141263
C 2.635926 -1.803634 -1.609792
H 3.732584 -1.738585 -1.535032
H 2.391480 -2.543284 -2.382196
H 2.265904 -0.830791 -1.955166
N 1.972486 -2.150516 -0.342435
H 2.245474 -1.462621 0.393664
H 2.296660 -3.063890 -0.021976
C -3.335274 -0.831465 2.052779
H -3.940189 -1.742543 2.133643
H -3.606889 -0.163690 2.885019

H	-3.612527	-0.328673	1.118636
N	-1.907833	-1.179672	2.005322
H	-1.622665	-1.633493	2.872652
H	-1.337892	-0.328902	1.899601
C	-1.465797	-2.790487	-1.395808
H	-1.325876	-3.777505	-0.943601
H	-2.536855	-2.585358	-1.483519
H	-1.024980	-2.789711	-2.397171
N	-0.819388	-1.757819	-0.562179
H	0.237414	-1.938705	-0.459420
H	-1.243308	-1.707412	0.411716
C	3.572566	1.028388	1.219913
H	4.579689	0.607775	1.332775
H	3.430226	1.263839	0.158458
H	3.530184	1.978564	1.775942
N	2.566495	0.039945	1.634808
H	1.623204	0.440235	1.525114
H	2.680439	-0.169325	2.626778

36

IV-e E[DF-MP2-F12] -760.8305626

C	-0.967686	2.159033	0.301117
C	0.538104	2.142915	-0.146159
O	-1.604773	1.076039	0.195231
O	-1.338047	3.275415	0.724536
O	1.133292	1.176824	-0.596550
O	1.091084	3.337092	0.047297
H	-0.831324	-0.277095	-0.343234
H	0.296548	3.840317	0.418835
C	4.347998	-0.889357	-1.097702
H	3.684723	-1.296295	-1.870995
H	4.922228	-0.065050	-1.551552
H	5.053568	-1.682433	-0.820977
N	3.548289	-0.503064	0.071539
H	4.154429	-0.115145	0.794279
H	2.887669	0.239734	-0.183881
C	-0.457921	-1.467677	-2.045469
H	-1.258075	-1.010877	-2.635600
H	0.482801	-0.960565	-2.276048
H	-0.379695	-2.528486	-2.304617
N	-0.746182	-1.310865	-0.606459
H	0.019460	-1.763074	-0.007592
H	-1.695216	-1.673484	-0.342772
C	-4.650880	-1.329193	-0.548787

H	-4.940894	-2.368725	-0.742990
H	-5.529003	-0.796104	-0.151479
H	-4.391740	-0.871201	-1.510437
N	-3.475516	-1.295983	0.333993
H	-3.714693	-1.682766	1.247038
H	-3.182121	-0.324462	0.494669
C	1.022375	-2.022042	2.406517
H	0.158682	-2.606151	2.747414
H	1.853052	-2.201636	3.106733
H	0.750935	-0.961623	2.471998
N	1.338779	-2.352226	1.007601
H	1.586116	-3.339284	0.930936
H	2.170570	-1.805660	0.702333

36
IV-f E[DF-MP2-F12] -760.8301225

C	0.357805	1.760471	-0.732791
C	1.633242	1.503193	0.158923
O	-0.454421	0.811128	-0.897393
O	0.317950	2.930591	-1.174128
O	1.913830	0.462227	0.736929
O	2.378211	2.599948	0.207787
H	-0.311569	-0.673704	-0.269171
H	1.814795	3.216225	-0.369595
C	-1.427650	-2.482145	-0.285109
H	-2.352539	-1.920841	-0.106119
H	-1.339583	-2.690460	-1.356041
H	-1.440775	-3.430220	0.262324
N	-0.278582	-1.658435	0.151934
H	-0.257090	-1.512920	1.198794
H	0.655364	-2.064739	-0.125438
C	-1.017352	0.097063	3.313776
H	-1.871260	-0.486855	3.675417
H	-0.714378	0.796306	4.108017
H	-1.359931	0.684100	2.454069
N	0.052039	-0.815519	2.876902
H	0.376986	-1.384073	3.658372
H	0.857464	-0.280545	2.539085
C	-4.285306	0.219138	-1.562700
H	-5.253809	-0.216180	-1.285274
H	-4.477313	1.179839	-2.070766
H	-3.824664	-0.453868	-2.296949
N	-3.421950	0.306742	-0.379949
H	-3.838181	0.939042	0.303593

H	-2.516808	0.714689	-0.638902
C	2.637636	-2.041358	-2.069514
H	2.352346	-2.951757	-2.609261
H	1.986170	-1.230598	-2.415953
H	3.672118	-1.786756	-2.346484
N	2.432573	-2.239366	-0.625292
H	2.683041	-1.385670	-0.117436
H	3.036240	-2.984287	-0.278526

36

IV-g E[DF-MP2-F12] -760.8296169

C	1.194166	-0.787932	0.290439
C	1.076868	0.790066	0.251839
O	2.391152	-1.330494	0.337007
O	0.180688	-1.483787	0.268001
O	2.084884	1.455508	-0.040371
O	-0.087061	1.229818	0.512472
H	3.214284	-0.671980	0.346300
H	-1.338283	0.304282	0.649155
C	-2.933288	-3.479280	-0.914842
H	-3.995682	-3.442524	-1.184255
H	-2.466172	-4.306040	-1.472896
H	-2.869414	-3.720528	0.152503
N	-2.312610	-2.168547	-1.151089
H	-1.324298	-2.180117	-0.871574
H	-2.335332	-1.941572	-2.145194
C	5.431647	-0.070485	-0.836055
H	5.954143	-1.021473	-0.680169
H	4.834765	-0.164311	-1.750033
H	6.183827	0.716557	-0.998691
N	4.531029	0.194945	0.290980
H	3.956350	1.033812	0.138427
H	5.044679	0.302875	1.164173
C	-2.839933	3.215901	-1.425773
H	-3.915984	3.350609	-1.589019
H	-2.328078	4.144942	-1.723265
H	-2.499252	2.419914	-2.098546
N	-2.593238	2.809639	-0.035258
H	-2.881599	3.551586	0.602191
H	-1.586351	2.662858	0.115707
C	-2.751899	-0.434697	2.052004
H	-2.541075	0.356073	2.778233
H	-3.813874	-0.696578	2.102191
H	-2.147282	-1.311626	2.300901

N -2.390622 0.027512 0.698931
H -2.860116 0.926686 0.438669
H -2.549843 -0.728093 -0.022287

36
IV-h E[DF-MP2-F12] -760.8294411
C 0.044347 2.064232 -0.428151
C 1.598020 1.967284 -0.210972
O -0.616443 0.987640 -0.362002
O -0.351911 3.227244 -0.649180
O 2.221552 0.945001 0.013164
O 2.154474 3.172594 -0.309918
H 0.075722 -0.483068 0.006049
H 1.340573 3.736706 -0.495089
C -3.061044 -1.872229 -1.948394
H -3.132397 -2.950822 -2.131466
H -3.329556 -1.686993 -0.901687
H -3.800984 -1.366416 -2.588279
N -1.674872 -1.426156 -2.168233
H -1.591434 -0.424758 -1.965224
H -1.412237 -1.549001 -3.146075
C -2.989530 0.635755 2.597658
H -1.994303 0.406050 2.999344
H -3.150924 1.720442 2.718917
H -3.722054 0.111928 3.224710
N -3.078299 0.149899 1.215472
H -2.394293 0.645167 0.631290
H -3.997787 0.374199 0.834850
C -0.412044 -2.100161 1.276389
H -0.404268 -3.194089 1.239164
H 0.191051 -1.767561 2.126731
H -1.439153 -1.733798 1.392733
N 0.156335 -1.546537 0.026190
H -0.422734 -1.815942 -0.812596
H 1.174213 -1.810545 -0.104616
C 3.761994 -2.473340 0.905398
H 4.840040 -2.253103 0.868065
H 3.370192 -2.063344 1.843283
H 3.635725 -3.561980 0.938468
N 2.996894 -1.916100 -0.220379
H 3.374133 -2.255699 -1.104992
H 3.078242 -0.894189 -0.233012

36

IV-i E[DF-MP2-F12] -760.8286288

C	0.036797	1.961141	-0.867953
C	1.196276	1.798009	0.185620
O	0.161842	3.118602	-1.505606
O	-0.850695	1.151638	-1.094209
O	1.978412	2.774748	0.189210
O	1.222925	0.742289	0.873674
H	0.993335	3.478401	-1.051405
H	0.319222	-0.613258	0.603799
C	-2.657091	-2.143465	-1.896419
H	-3.286182	-1.806288	-2.734618
H	-2.706097	-3.237858	-1.851084
H	-3.084675	-1.749651	-0.966910
N	-1.251945	-1.714440	-1.992975
H	-0.833518	-2.058862	-2.856821
H	-1.197801	-0.691009	-2.020616
C	-0.683757	-2.193527	1.609266
H	-0.180903	-2.005313	2.562786
H	-1.653613	-1.681573	1.597467
H	-0.824036	-3.272072	1.484817
N	0.144834	-1.661813	0.503372
H	-0.313261	-1.821432	-0.440679
H	1.127709	-2.036363	0.526702
C	3.863513	-1.808402	-0.411892
H	4.070437	-2.858542	-0.649529
H	3.337764	-1.370617	-1.268674
H	4.825094	-1.282627	-0.306182
N	3.000279	-1.728242	0.776018
H	2.790797	-0.745210	0.990493
H	3.484733	-2.110938	1.587741
C	-2.674095	1.083180	2.298772
H	-2.887450	2.162648	2.220381
H	-3.264791	0.681996	3.131844
H	-1.614952	0.975474	2.562466
N	-2.949485	0.311483	1.079056
H	-2.375800	0.671365	0.311401
H	-3.921516	0.442969	0.799396

36

IV-j E[DF-MP2-F12] -760.8286281

C	0.036445	1.961150	-0.868012
C	1.196010	1.798113	0.185408
O	0.161334	3.118580	-1.505742
O	-0.851049	1.151593	-1.094067

O 1.978140 2.774876 0.188856
O 1.222802 0.742471 0.873559
H 0.992866 3.478516 -1.051600
H 0.319351 -0.613143 0.603333
C -0.683146 -2.193521 1.609019
H -0.179989 -2.005270 2.562371
H -1.653033 -1.681619 1.597548
H -0.823386 -3.272073 1.484615
N 0.145069 -1.661723 0.502864
H 1.127999 -2.036120 0.526037
H -0.313220 -1.821444 -0.441089
C -2.657339 -2.143756 -1.896127
H -2.706156 -3.238152 -1.850642
H -3.084780 -1.749915 -0.966565
H -3.286696 -1.806797 -2.734214
N -1.252300 -1.714504 -1.993067
H -1.198261 -0.691066 -2.020525
H -0.834045 -2.058751 -2.857062
C 3.864047 -1.808116 -0.411772
H 4.070972 -2.858248 -0.649444
H 3.338536 -1.370236 -1.268650
H 4.825635 -1.282408 -0.305790
N 3.000528 -1.728001 0.775928
H 2.790960 -0.744994 0.990410
H 3.484739 -2.110764 1.587762
C -2.673650 1.082863 2.299318
H -3.264112 0.681609 3.132518
H -1.614431 0.975159 2.562686
H -2.887063 2.162323 2.221030
N -2.949366 0.311218 1.079609
H -2.375927 0.671221 0.311821
H -3.921491 0.442690 0.800256

36
IV-k E[DF-MP2-F12] -760.8264762
C 0.296011 -1.463311 0.672555
C -0.273288 -1.410683 -0.770420
O -0.378621 -0.789035 1.546397
O 1.374159 -2.046304 0.894672
O -1.346106 -1.985099 -1.035461
O 0.393989 -0.665028 -1.590384
H -1.732805 -0.452101 1.069770
H 1.747276 -0.352208 -1.091335
C 1.566778 2.459983 1.935403

H	0.845211	2.639312	1.129178
H	1.071600	2.703572	2.889065
H	2.399576	3.159991	1.796098
N	2.051658	1.076435	1.846747
H	2.714063	0.885885	2.599013
H	1.269184	0.419491	1.983057
C	-1.627728	2.561795	-1.791299
H	-2.478682	3.229979	-1.611349
H	-0.905810	2.719697	-0.980880
H	-1.146138	2.868158	-2.733722
N	-2.073315	1.162445	-1.772157
H	-1.274951	0.535189	-1.950287
H	-2.737442	0.993342	-2.528067
C	-3.826646	0.013606	1.516152
H	-3.618702	0.986846	1.971719
H	-4.788522	0.064800	0.994526
H	-3.889881	-0.736430	2.310395
N	-2.739963	-0.333136	0.586623
H	-2.619168	0.351632	-0.217859
H	-2.843762	-1.249420	0.124650
C	3.845644	0.131030	-1.495911
H	3.645594	1.129932	-1.896091
H	4.803976	0.146855	-0.965589
H	3.910849	-0.572539	-2.331415
N	2.750885	-0.263967	-0.595613
H	2.625367	0.371961	0.246412
H	2.846321	-1.207181	-0.187173
